# Roles of noncoding RNAs in preeclampsia

**DOI:** 10.1186/s12958-021-00783-4

**Published:** 2021-07-02

**Authors:** Ningxia Sun, Shiting Qin, Lu Zhang, Shiguo Liu

**Affiliations:** 1 Department of Medical Genetic, The Affiliated Hospital of Qingdao University, 16 Jiangsu Road, Qingdao, 266003 China; 2 Department of Gynecology and obstetrics, The Affiliated Hospital of Qingdao University, Qingdao, 266003 China; 3 Department of Cardiology, The Affiliated Hospital of Qingdao University, Qingdao, 266003 China; 4 Prenatal Diagnosis Center, The Affiliated Hospital of Qingdao University, Qingdao, 266003 China

**Keywords:** microRNA, lncRNA, circRNA, Biomarker, Preeclampsia

## Abstract

Preeclampsia (PE) is an idiopathic disease that occurs during pregnancy. It comprises multiple organ and system damage, and can seriously threaten the safety of the mother and infant throughout the perinatal period. As the pathogenesis of PE is unclear, there are few specific remedies. Currently, the only way to eliminate the clinical symptoms is to terminate the pregnancy. Although noncoding RNA (ncRNA) was once thought to be the “junk” of gene transcription, it is now known to be widely involved in pathological and physiological processes, including pregnancy-related disorders. Moreover, there is growing evidence that the unbalanced expression of specific ncRNA is involved in the pathogenesis of PE. In the present review, we summarize the expression patterns of ncRNAs, i.e., microRNAs (miRNAs), long noncoding RNAs (lncRNAs), and circular RNAs (circRNAs), and the functional mechanisms by which they affect the development of PE, and examine the clinical significance of ncRNAs as biomarkers for the diagnosis of PE. We also discuss the contributions made by genetic polymorphisms and epigenetic ncRNA regulation to PE. In the present review, we wish to explore and reinforce the clinical value of ncRNAs as noninvasive biomarkers of PE.

## Introduction

Preeclampsia (PE) is a pregnancy-related disorder that is associated with the unprecedented onset of hypertension (systolic blood pressure ≥ 140 mmHg, diastolic blood pressure ≥ 90 mmHg). It occurs after the 20th week of gestation, and frequently near term. It is estimated that PE occurs in 3–8% of pregnant women globally [1]. Although PE is usually identified by new episodes of hypertension and proteinuria after 20 weeks of gestation, pregnant women without proteinuria may be diagnosed with the disorder if they present with one of the following: thrombocytopenia (a platelet count of less than 100,000 per μL); impaired liver function, such as an abnormal rise in the blood concentration of transaminase (to twice the normal concentration), or renal insufficiency (a serum creatinine concentration greater than 1.1 mg/dL, or in the absence of other kidney disease, doubling of the serum creatinine concentration); pulmonary edema; and the unprecedented onset of headaches that are unresponsive to medication and cannot be accounted for by an alternative diagnoses or visual symptoms [1]. Currently, a strategy for the timely detection and diagnosis of PE is urgently needed. This would avoid emergencies or existing complications with target organs. There are several related theories about the causes of PE, including chronic uterine or placental ischemia, immune disorders, genetic imprinting [2], trophoblast apoptosis and necrosis [3], and excessive trophoblast tolerance to inflammatory reactions [4]. Moreover, previous observations have indicated that an imbalance of angiogenesis factors may also play an important role in the pathogenesis of PE [5]. All these pathogenesis processes may be affected by genetic, epigenetic, environmental, and physiological factors, and there is growing evidence that epigenetics play a role in PE [6]. With regard to epigenetics, modifications to both DNA and histones are intermediately involved in the regulation of gene activity. Furthermore, the regulation of functional noncoding RNAs (ncRNAs) can alter gene activity, which modulates gene expression and transcription, chromatin structure, epigenetic memory, selective RNA splicing, and protein translation [7].

ncRNA is a type of functional RNA molecule that is not usually translated into protein. It accounts for 98% of the human genome, and includes housekeeping ncRNA (transfer RNA (tRNA), ribosomal RNA (rRNA), and small nuclear RNA (snRNA)) and regulatory ncRNA (small interfering RNA (siRNA), microRNA (miRNA), piwi-interacting RNA (piRNA), long noncoding RNA (lncRNA), and circular RNA (circRNA)) [8]. The regulatory ncRNAs can be divided into three types—lncRNA (> 200 nucleotides (nt)), miRNA (< 200 nt), and circRNA (circular structure)—which can regulate cell processes through direct interaction with each other [9]. For example, miRNAs can regulate gene expression by targeting mRNA [10] or by adjusting its stability by targeting circRNAs and lncRNAs [11–13]. Alternatively, circRNAs and lncRNAs can serve as “sponges” to adjust the availability of miRNAs [14, 15]. ncRNAs can also adjust the physiological function of cells by interacting with DNA or proteins [9, 16] The aberrant expression of ncRNAs or their abnormal interactions can lead to the development of various diseases, including PE and cardiovascular disease. There is increasing evidence that miRNAs, lncRNAs, and circRNAs are widely involved in the pathogenesis of PE. In the present review, we summarize the role of these transcripts in the pathogenesis of PE, and highlight the possible use of ncRNA as a noninvasive tool for diagnosing the condition.

## miRNAs and preeclampsia

miRNAs are the best studied of the ncRNAs. They are small single-stranded structures of approximately 22–25 nucleotides that can act as regulators at the post-transcriptional level. When miRNA molecule binds to the 3′-untranslated region of mRNA molecule, it induces the degradation of the mRNA or prevents its translation. It is estimated that miRNAs are able to regulate the translation of more than 60% of the protein-coding genes involved in many physiological processes, such as proliferation, differentiation, apoptosis, and development [17]. Numerous studies have revealed abnormal miRNA expression in the placentas or peripheral blood of patients with PE. Aberrant miRNAs can target downstream genes and reduce the migration and invasion of trophoblasts, or increase cell apoptosis, ultimately resulting in PE.

### Expression pattern of miRNAs in patients with PE

To date, approximately 70 miRNAs have been reported to be differentially expressed in PE tissues. Table 1 summarizes the differentially expressed miRNAs involved in the pathogenesis of PE. For the first time, Pineles et al. reported seven differentially expressed miRNAs in the placentas of patients with PE who gave birth to either normal babies or babies that were small for their gestational age. Among these miRNAs, only the levels of miR-182 and miR-210 increased significantly relative to the corresponding levels experienced during normal pregnancy [118]. This finding presented new targets for the pathophysiology of PE. In view of the role of miR-210 in PE, related studies with larger sample sizes further confirmed that the placental expression of miR-210 in patients with PE did indeed increase significantly compared to the corresponding levels experienced during normal pregnancy [18–20]. miR-210 can inhibit the proliferation, invasion, and angiogenesis of trophoblasts by acting on the downstream target genes that encode KCMF1 [20], NOTCH1 [21] and MAPK [18]. Upregulated levels of miR-210 have also been detected in peripheral blood serum [18]. It has also been reported that the disturbed expression of miR-182-5p can inhibit trophoblast proliferation, invasion, and migration by acting on the 3′-untranslated region of *RND3* [24]. Zhang et al. first reported that miR-155, an inflammation-related miRNA, is overexpressed in the placentas of PE patients, and is involved in the pathogenesis of PE because it downregulates *CYR61* [25]. miR-155 can bind the target genes that encode cyclin D1 [16] and eNOS [26] to affect the migration and proliferation of trophoblasts. Differentially expressed miRNAs have been found in exosomes [50, 64, 103, 112] and Mesenchymal stem cells (MSCs) [28, 51, 71, 76] as well as in placental tissues and peripheral serum or plasma. For example, miR-16 upregulation was first confirmed in the placentas of patients with PE [29]. Studies have shown that miR-16 is differentially expressed in the decidual MSCs of patients with severe PE and normal patients, and can inhibit the proliferation and migration of decidual-derived MSCs by targeting cyclin E1 and inducing cell cycle arrest [28]. More interestingly, miR-16 overexpression in decidual-derived MSCs can also reduce the ability of human umbilical vein endothelial cells to form blood vessels [28].

**Table 1:** Dysregulation of miRNAs in PE

MiRNA	Sample type	Status	Targets	Function	Ref.
miR-210	Serum/placenta	upregulated	NOTCH1/PTPN2/THSD7A/KCMF1/FOXA1	Upregulation of miR-210 decreased NOTCH1/PTPN2/THSD7A/KCMF1 expression, impaired HTR-8/SVneo proliferation, migration, invasion, and tube-like formation capabilities, and promoted apoptosis.	[18–23]
miR-182-5p	placenta	upregulated	RND3	the increased miRNA-182-5p expression could inhibit the migratory and invasive ability of trophoblast cells through targeted degrading RND3 protein	[24]
miR-155	placenta/placenta-associated serum exosomes	upregulated	CYR61/Cyclin D1	Overexpression of miR-155 in HTR-8/SVneo cells inhibited cell invasion, proliferation and increased cell number at the G1 stage in trophoblast cells	[16, 25]
miR-155-5p	placenta	upregulated	eNOS	miR-155 inhibited cell invasion in trophoblast cells, and the effect was rescued by over expression of eNOS.	[26]
miR-195	placenta	downregulated	ActRIIB/ActRIIA	miR-195 could promote cell invasion via directly targeting ActRIIB/ActRIIA in human trophoblast cells	[27]
miR-16	placenta/mesenchymal stem cell (MSC)	upregulated	CCNE1 /VEGF-A/Notch2	over-expressed miR-16 inhibited the proliferation and migration of decidua-derived mesenchymal stem cells /BeWo and JEG-3 cells, and induced cell-cycle arrest by targeting cyclin E1	[28, 29]
miRNA-376c	placenta/plasma/exosome	downregulated	ALK5/ALK7/25-OH-VD	miR-376c inhibits both ALK5 and ALK7 expression to impair transforming growth factor-β/Nodal signaling, leading to increases in cell proliferation and invasion	[30, 31]
miR-29b	decidua-derived mesenchymal stem cell (dMSC)/placenta	upregulated	MMP2/MCL1/ VEGFA/ ITGB1/HDAC4	miR-29b induced apoptosis and inhibited invasion and angiogenesis of trophoblast cells.	[32]
miR-101	placenta	downregulated	ERp44/BRD4/CXCL6	miR-101 could promote apoptosis and inhibite the proliferation and migration of trophoblasts	[10, 33, 34]
miR-18a	placenta/plasma	downregulated	Smad2/ER1/ESRα	miR-18a could promote trophoblast cell invasion and suppress apoptosis of human trophoblast cells	[35–37]
miR-20a	placenta	upregulated	Foxa1/VEGF	the upregulated miR-20a in human preeclampsia tissue can inhibit the proliferative and invasive activities of trophoblast cells	[23, 38]
miR-125b-1-3p	placenta	upregulated	S1PR1	miR-125b-1-3p inhibited the invasiveness of human trophoblast cells,	[39]
miR-125b	placenta/plasma	upregulated	SGPL1/ STAT3/KCNA1 /GPC1/Trop-2	upregulated miR-125b can impair endothelial cell function, reduce cell proliferation and invasion and migration	[40–43]
miR-126	placenta	downregulated	VEGF/ PIK3R2	miR-126 enhanced endothelial progenitor cell (EPC) proliferation, differentiation and migration	[44, 45]
miR-196b	plasma	downregulated	/	/	[46]
miR-206	plasma	upregulated	VEGF/IGF-1/ET-1	miR-206 modulated trophoblast cells migration and invasion	[47–49]
miR-494	Mesenchymal stem cells(MSC)/serum exosomes	upregulated	VEGF/CDK6	supernatant from miR-494-overexpressing dMSCs could reduce HTR-8/SVneo migration, impair HUVEC capillary formation and arrest G1/S transition	[50, 51]
miR-519d-3p	placenta	upregulated	MMP-2	upregulation of miR-519d-3p may contribute to the development of PE by inhibiting trophoblast cell migration and invasion via targeting MMP-2	[52]
miR-335	placenta	upregulated	eNOS/Sp1	miR-335-5p could inhibit transforming from epithelial to mesenchymal and cell migration	[53, 54]
miR-584	placenta	upregulated	eNOS	miR-335 inhibited the migratory ability of the trophoblast cells, and the effect was ‘rescued’ by overexpressed eNOS	[53]
miR-34a	placenta	upregulated	MYC/BCL-2, Notch-1	Overexpression of miR-34a inhibited cell proliferation, migration and invasion, and induced trophoblast cell apoptosis by inhibiting expression of BCL-2 protein	[55–57]
miR-204	placenta	upregulated	MMP-9	miR-204 may contribute to the development of preeclampsia by inhibiting trophoblastic invasion	[58]
miR-193b-3p	placenta	upregulated	TGF-β2	miR-193b-3p could regulate trophoblasts migration and invasion through binding onto the 3’UTR target site of TGF-β2	[59]
miR-193b-5p	placenta	upregulated	APLN/FGF13	Overexpression of miR-193b-5p inhibited trophoblast cell proliferation and migration	[60]
miR-203	placenta	upregulated	VEGFA/SOCS-3	miR-203 overexpression inhibited the proliferation, migration and invasion ability of HTR-8/SVneo cells, meanwhile which increased the endothelial inflammatory response	[61–63]
miRNA-203a-3p	placental mononuclear cells and exosomes	downregulated	IL24	microRNA-203a-3p was able to suppress the proliferation capacity of LPS-stimulated mononuclear macrophages, and it exerted anti-inflammatory effects via down-regulating IL24 in THP-1 cells.	[64]
miR-885-5p	placenta	upregulated	MMP-9	/	[65]
miR-141	placenta/plasma	upregulated	EG-VEGF/ CXCL12β/ CXCR2 / 4	miR-141 could inhibit the invasion and vascularization abilities, and promote the apoptosis of HTR-8/SVneo cells	[66, 67]
miR-141-5p	placenta	downregulated	ATF2	miR-141-5p up-regulated transcription factor ATF2 to promote phosphatase DUSP1 expression, which reduces p-MAPK1 and ERK1/2 expression to promote preeclampsia.	[68]
miR-128a	placenta	upregulated	Bax	miR-128a induced the apoptosis of HTR-8/SVneo cells by down-regulating Bax through the mitochondrial apoptosis pathway.	[69]
miR-145	placenta	downregulated	PI3K/MUC1	miR-145 may serve key roles in the regulation of trophoblast cell proliferation and invasion	[70]
miR-136	Mesenchymal stem cells (MSCs)/serum exosomes	upregulated	BCL2/VEGF	MiR-136 significantly increase the apoptosis and suppress the proliferation of MSCs, and it could also inhibit the capillary formation and trophoblast cell invasion.	[71]
miR-520 g	serum	upregulated	MMP-2	Elevated maternal serum level of miR-520 g level in first trimester could suppress the migration and invasion of trophoblast, and might play a role in the defective spiral artery remodeling,	[72]
miR-20b	placentas and peripheral blood	upregulated	MMP-2	miR-20b inhibited trophoblastic invasion by targeting MMP2	[73]
miR-23a	placenta	upregulated	XIAP/HDAC2	miR-23a reduced HTR-8/SVneo cell migration and invasion and increased HTR-8/SVneo cell apoptosis	[74, 75]
miR-495	peripheral blood exosomes/umbilical cord mesenchymal stem cells (UCMSCs)	upregulated	Bmi-1	The supernatants from miR-495-overexpressed inhibited the migration of MSCs and HTR-8/SVneo, invasion of HTR-8/SVneo and tube formation of HUVEC	[76]
miR-137	placenta	upregulated	ERRa	MiRNA-137 significantly reduced the proliferation and migration of placenta trophoblast cells	[77]
miR-93	placenta/plasma	upregulated	MMP-2	miR-93 reduced migration and invasion of immortalized trophoblast cells.	[78]
miR-144	placenta	downregulated	PTEN	miR-144 induced significant increase in cell proliferation, migration, invasion, and decrease in cell apoptosis, and also affected the cell cycles	[79]
miR-144-3p	placenta	downregulated	Cox-2	Downregulation of miR-144-3p led to systemic inflammation and endothelial cell injury	[80]
miR-942	plasma	downregulated	ENG	Decreased miR-942 expression decreased the invasive ability of TEV-1 cells, and inhibited the HUVEC angiogenesis assay	[81]
miR-134	placenta	upregulated	ITGB1	MiR-134 suppressed the infiltration of trophoblast cells by targeting ITGB1	[82]
miR-362-3p	placenta	upregulated	Pax3	miR-362-3p/Pax3 axis regulates cell viability, migration and invasion of HTR8/SVneo cells under hypoxia.	[83]
miR-454	placenta	downregulated	EPHB4/ALK7	MiR-454 promotes the proliferation and invasion of trophoblast cells, and inhibit the apoptosis	[84, 85]
miR-30a-3p	placenta	upregulated	IGF-1	the over-expression of miR-30a-3p alter the invasive capacity of JEG-3 cells and induce the apoptosis of HTR-8/SVneo cells	[86]
miR-31-5p	serum	upregulated	eNOS	NF-κB-responsive miR-31-5p elicits endothelial dysfunction, hypertension, and vascular remodeling via post-transcriptional down-regulation of eNOS	[87]
miR-423–5p	plasma	upregulated	IGF2BP1	MiR-423-5p inhibited migration, invasion and proliferation as well as induced apoptosis in HTR-8/SVneo cells.	[88]
miR-299	placenta	upregulated	HDAC2	miR-299 suppressed the invasion and migration of trophoblast cells partly via targeting HDAC2	[89]
miR-4421	placenta	upregulated	CYP11B2	overexpression of miR-4421 inhibited trophoblast proliferation and significantly blocked cell cycle	[90]
miR-320a	placenta	upregulated	IL-4/ERRγ	Excessive miR-320a expression remarkably suppressed trophoblast invasion and proliferation	[12, 91]
miR-517-5p	placenta	upregulated	MMP-2	MiR-517-5p could promote proliferative and invasive abilities of JAR cells by inhibiting ERK/MMP-2 pathway	[92]
miR-517c-3p	plasma	upregulated	TNFSF15	miR-517c-3p could suppress cell growth and proliferation, and promote placental hypoxia, immune response and apoptosis.	[93]
miR-let-7d	placenta	upregulated	/	low expression levels of miR-let-7d plays a central role in suppressing apoptosis in addition to promoting the proliferation and invasion of PE TCs.	[94]
miR-218-5p	placenta	downregulated	TGF-β2	miR-218-5p accelerated spiral artery remodeling in a decidua-placenta co-culture and promoted trophoblast invasion and enEVT differentiation	[95]
miR-181a-5p	placenta	upregulated	IGF2BP2	miR-181a-5p may trigger antiproliferation and inhibition of cell cycle progression, induce apoptosis, and suppress invasion in HTR-8/SVneo and JAR cells	[96]
miR-142-3p	placenta	upregulated	TGF-β1	miR-142-3p suppressed cell invasion and migration by reactivating the TGF-β1/Smad3 signaling pathway.	[97]
miR-145-5p	placenta	downregulated	FLT1/Cyr61	miR-145-5p promoted trophoblast cell growth and invasion	[98, 99]
miR-30b	placenta	upregulated	MXRA5	miR-30b might contribute to PE through inhibiting cell viability, invasion while inducing apoptosis of placental trophoblast cells via MAPK pathway by targeting MXRA5.	[100]
miR-454	placenta	downregulated	ALK7/EPHB4	miR-454 promotes the proliferation and invasion of trophoblast cells by inhibiting EPHB4 /ALK7	[84, 85]
miR-200a	plasma	upregulated	EG-VEGF/TTR	miR-200a suppressed primary cilia formation and inhibited trophoblast invasion.	[101, 102]
miR-548c-5p	placenta/ serum exosome	downregulated	PTPRO	miR-548c-5p could inhibit the proliferation and activation of LPS-stimulated macrophages and decrease levels of inflammatory cytokines	[103]
miR-342-3p	placenta	upregulated	PDGFRA/ID4	miR-342-3p was proposed to inhibit the proliferation, migration, invasion and G1/S phase transition of HTR8/SVneo cells	[104, 105]
miR-431	placenta	upregulated	ZEB1	miR-431 inhibited the migration and invasion of trophoblastic cells	[106]
miR-221-3p	placenta	downregulated	THBS2	miR-221-3p overexpression inhibited apoptosis, increased cell population at S phase, and decreased cell population at G0/G1 phase of HTR-8/SVneo cells	[107]
miR-152	placenta	upregulated	VEGF	the increased miR-152 expression can promote the apoptosis of trophoblast cells.	[108]
miR-133	serum	upregulated	Rho/ROCK	MiR-133 may affect the apoptosis of trophoblasts in the placenta tissues	[109]
miR-384	placenta/plasma	upregulated	PTBP3	Cell proliferation and migration were inhibited by miR-384 overexpression	[110]
miR-21	Serum/placenta	upregulated	FOXM1	MiR-21 may regulate placental cell proliferation	[111]
miR-125a-5p	placenta/exosome	upregulated	VEGFA	miR-125a-5p might affect HTR8/SVneo cell proliferation and migration and inhibit angiogenesis	[112]
miR-214-3p	serum	upregulated	PIGF	Downregulation of miR-214-3p promoted trophoblast invasion into the decidua, as well as spiral artery remodeling	[113]
miR-518b	plasma	upregulated	EGR1	Increased miR-518b inhibited trophoblast migration and angiogenesis	[114]
miRNA-646	serum	upregulated	VEGF-A	miR-646 suppressed endothelial progenitor cells (EPCs) multiplication, differentiation and migration.	[115]
miR-215-5p	placenta	upregulated	CDC6	miR-215-5p inhibited both the migration and invasive potential of trophoblasts, besides decreasing the G1-S transition in HTR-8/SVneo cells	[116]
miR-483	venous blood/ umbilical cord blood / placental tissue	downregulate	IGF1	miR-483 regulates the expression of PI3K, Akt, and mTOR in endothelial progenitor cells	[117]

### Diagnostic value of miRNAs in PE

Numerous studies have confirmed that miRNAs are involved in the pathogenesis of PE and are differentially expressed in patients with this disease. Some researchers have assessed the diagnostic value of miRNA with regard to PE by drawing receiver operating characteristic (ROC) curves. For example, Zhang et al. showed that the levels of miR-942 decreased significantly in the plasma of patients with PE compared to the corresponding levels in normal patients, and had 65.4% sensitivity and 69.2% specificity with regard to PE diagnosis [81]. Table 2 summarizes the results of research into the value of miRNAs with regard to the diagnosis of PE.

**Table 2:** Diagnostic value of miRNAs in PE

MiRNA	Sample type	Area under curve	Sensitivity	Specificity	Ref.
miR-31-5p	serum	0.96	95.65%	92.39%	[113]
miR-155-5p	serum	0.931	89.13%	88.04%	[113]
miR-214-3p	serum	0.924	90.22%	79.35%	[113]
miR-1290-3p	serum	0.957	94.57%	84.78%	[113]
miR-210	serum	0.75	/	/	[119]
miR-155	serum	0.718	/	/	[119]
miR-206	plasma	0.94	97%	77.50%	[48]
miR-518b	plasma	0.715	/	/	[120]
miR-31	plasma	0.875	95.00%	70.00%	[121]
miR-21	plasma	0.793	65.10%	90.30%	[121]
miRNA-136	exosome	1	95%	100%	[50]
miRNA-494	exosome	0.868	86%	95%	[50]
miRNA-495	exosome	0.94	90%	83%	[50]
miR-195	placenta	0.82	80%	80%	[122]
miR-423-5p	plasma	0.844	87.50%	80%	[123]
miR-942	plasma	0.718	65.40%	69.20%	[81]
miR-517-5p	plasma	0.7	42.90%	86.20%	[124]
miR-516-5p	plasma	0.608	38.10%	84.50%	[124]
miR-518b	plasma	0.55	34.40%	78.70%	[124]

### Association between miRNA variants and the risk of PE

Given that genetic factors play important roles in the occurrence of PE, several studies have focused on the relationship between single nucleotide polymorphisms (SNPs) in miRNAs and the risk of PE. It has also been reported that the miR-146a rs2910164 polymorphism may not be associated with PE susceptibility, cytokines, or related characteristics in black women from South Africa, whereas the GC/CC genotype may reduce susceptibility to severe PE [125]. Interestingly, Salimi et al. discovered that a maternal/placental miR-146a polymorphism (rs2910164) was associated with PE or risk of severe PE, after they genotyped it in the blood samples and placentas from the Asian mainland, using polymerase chain reaction (PCR)–fragment length polymorphism [126]. Table 3 summarizes the reported results.

**Table 3:** Association between polymorphisms with SNPs and risk of PE

MiRNA	Sample type	Risk Variant	Association with PE	minor allele frequencyn n(%)	Ref.
miRNA-155	serum	rs767649	A allele	T allele(39.2)	[127]
miRNA-146a	maternal blood	rs2910164	Negative	C allele(38.7)	[126, 128]
miRNA-27a	maternal blood/Placental	rs895819	GC + CC vs GG	T allele(46)	[129]
miRNA-196a2	maternal blood	rs11614913	Negative	T allele(38.6)	[130]
miR-499	Placental	rs3746444	CT + TT vs CC	C allele(37.3)	[130]
miRNA-152	maternal blood	rs12940701	CC vs TC + TT	T allele(28.9)	[131]
miRNA-26a	maternal blood/Placental	rs7372209	Negative	T allele(16)	[132]

### Demethylation of the miRNA in PE

In addition to the previously reported abnormal expression of miRNA in PE patients, some studies also found that the methylation level of some abnormal expression of miRNA is associated with the risk of PE. Rezaei et al. found that hypomethylation of the miR-34a promoter was associated with the occurrence and severity of PE, when they applied methylation-specific PCR to investigate samples from 104 pregnant women with PE and 119 normotensive pregnant women [133]. Moreover, studies have shown that the abnormal regulation of the miR-let-7 family is related to PE. The methylation status of miR-let-7a in PE was evaluated by methylation-specific PCR and bisulfite sequencing PCR analyses. The results suggested that hypomethylated miR-let-7a promotes PE by downregulating Bcl-xl and YAP1 [134]. The miR-510 promoter region in bisulfite-treated PE DNA samples was also found to be unmethylated, compared to the corresponding region in the control samples [135].

## lncRNAs and PE

lncRNAs are composed of more than 200 nucleotides. They promote the development of human diseases by participating in various biological processes, including genomic imprinting, chromatin modification, regulation of transcription and post-transcriptional gene expression, nuclear transport, and other regulatory processes [136]. There is abundant evidence to suggest that lncRNA expression in the placenta and peripheral blood differs between healthy pregnant women and patients with PE. This indicates that abnormal lncRNA expression is involved in the pathogenesis of PE.

### Expression pattern of lncRNAs in patients with PE

Table 4 summarizes the differentially expressed lncRNAs that participate in the pathogenesis of PE. For example, Liu et al. discovered that the levels of *GASAL1* lncRNA were downregulated in the placental tissues of 30 patients with PE, compared to the corresponding levels in 30 normal controls. They further demonstrated that *GASAL1* lncRNA can directly bind to functional RNA-binding protein SRSF1 to promote trophoblastic proliferation and progression from the G1 to the S phase through the mTOR and EBP1 signaling pathways. It can also inhibit trophoblastic apoptosis by downregulating cleaved caspase-3 and upregulating Bcl-2 [174]. Another well-known lncRNA, that of *MEG3*, is expressed in a variety of normal tissues, but is absent in many tumors and tumor cell lines [189]. Yu et al. found that the expression of lncRNA MEG3 in PE placental tissue decreased significantly by 72% compared with that in the normal controls, and MEG3 interruption induced the expression of E-cadherin but reduced that of N-cadherin. This confirmed that MEG3 inhibits trophoblastic migration and invasion. They also found that MEG3 downregulation affects the TGF-β/Smad pathway by inhibiting Smad7 expression, thereby suppressing epithelial–mesenchymal transition [147]. Wang and Zou also showed that by regulating the expression of NOTCH1, MEG3 can promote the apoptosis of trophoblasts, and inhibit their migration and invasion, thereby inducing PE [148].

**Table 4:** Dysregulation of lncRNAs in PE

LncRNA	Sample type	Status	Targets	Function	Ref.
BC030099	the whole blood (WB)	upregulated	/	/	[137]
LOC391533	placenta	upregulated	sFlt-1	This protein plays an important role in angiogenesis and vasculogenesis.	[138]
LOC284100	placenta	upregulated	/	/	
CEACAMP8	placenta	upregulated	/	/	
HOTAIR	placenta	upregulated	miR-106a	High level of HOTAIR represses the proliferation, migration and invasion of trophoblast cells through downregulating miR-106 in an EZH2-dependent manner.	[139]
		downregulated	PUM1/HOTAIR	HOTAIR promotes trophoblast invasion by activating PI3K-AKT signaling pathway.	[140]
AGAP2-AS1	placenta	downregulated	JDP2	AGAP2-AS1 knockdown could inhibit trophoblasts proliferation, invasion and spiral artery remodeling and promote cell apoptosis.	[141]
HOXA11-AS	placenta	downregulated	miR-15b-5p/HOXA-7/Lsd1 and Ezh2/RND3	Down-regulated HOXA11-AS inhibits trophoblast cell proliferation, migration and invasion, and promoted cell accumulation in G0–G1 phase and apoptosis.	[142]
TUG1	placenta	downregulated	miR-29b/MCL1/VEGFA /MMP2	TUG1 could act as a molecular sponge for miR-29b, thus down-regulating MCL1, VEGFA, and MMP2 to promote cell proliferation, invasion, and angiogenesis, while negatively regulated cell apoptosis.	[14]
			Ezh2/RND3	Down-regulated TUG1 inhibits trophoblast cell proliferation, migration, invasion and the formation of capillary-like networks and promotes trophoblast cell apoptosis.	[143]
			miR-204-5p	Down-regulated TUG1 negatively regulates trophoblast migration and invasion partly through sponging miR-204-5p and negatively regulating the expression and function of miR-204-5p.	[144]
SPRY4-IT1	placenta	upregulated	Bax/Bcl-2	SPRY4-IT1 overexpression significantly decreases the cell migration, proliferation and spiral artery remodeling, while increases cell apoptosis.	[145]
			Wnt/β-catenin pathway	Overexpression of SPRY4-IT1 suppresses trophoblast cell migration, invasion and spiral artery remodeling by inducing E-cadherin and β-catenin expression and decreasing vimentin expression.	[146]
MEG3	placenta	downregulated	TGF-β/ /E-cadherin/N-cadherin	Down-regulated MEG3 induces E-cadherin upregulation and N-cadherin and slug downregulation in HTR-8/SVneo cells, which inhibits trophoblast cell proliferation, migration, invasion and EMT.	[147]
			Notch1	Down-regulation of MEG3 could downregulate Notch1 expression to suppress trophoblast cells migration, invasion and promote its apoptosis.	[148]
			NF-κB/Caspase-3/ Bax	Down-regulation of MEG3 increases apoptosis and decreases migration of trophoblast cells by influencing expression of NF-κB, Caspase-3, and Bax protein expressions.	[149]
H19	placenta	downregulated	NOMO1/miR-675	Lowered expression of H19 participate in the excessive proliferation of trophoblast cells by downregulating miR-675 which targets NOMO1 and interferes with Nodal signaling.	[150]
			miR-148a-5p/P28/miR-216-3p/EBI3	Down-regulated could up-regulate the expression of miR-148-5p/miR-216-3p and the expressions of subunits of IL-27, P28 and EBI3 were thus suppressed.	[151]
			miRNAlet-7/the type III TGF-β receptor (TβR3)	H19 repression decreases TGF-β signaling via the Par6/Smurf1/RhoA pathway activated by TβR3, leading to impaired migration and invasion of EVT cells.	[152]
		upregulated	PI3K/AKT/mTOR pathways	LncRNA-H19 overexpression reduces cell viability but increases invasion and autophagy in trophoblast cells by enhancing phosphorylated levels of key kinases in the PI3K/AKT/mTOR pathways.	[153]
MIR503HG	placenta	upregulated	the matrix metalloproteinase-2/− 9, the snail /E-cadherin/ NF-κB signaling pathway	MIR503HG overexpression suppresses cell proliferation, invasion, and migration, and induces apoptosis and causes cell cycle arrest at the G0/G1 phase of HTR-8/SVneo	[154]
DLX6-AS1	placenta tissues	upregulated	miR-149–5p/ERP44 pathway	DLX6-AS1 inhibits proliferation and invasion of trophoblast cells, and suppresses angiogenesis of HUVEC cells by binding miR-149–5p/ERP44 pathway.	[11]
			miR-376c/ GADD45A	DLX6-AS1 may contribute to preeclampsia by impairing proliferative, migratory and invasive abilities of trophoblasts via the miR-376c/GADD45A axis.	[155]
ZEB2-AS1	placenta	downregulated	miR-149 / PGF	ZEB2-AS1 contributes to PE progression by inhibiting cell proliferative, migratory, invasive capacities and EMT via the miR-149/PGF axis in HTR-8/SVneo cells.	[156]
PRNCR1	placenta	upregulated	MAPK	Overexpression of LncRNA PRNCR1 in PE patients reduces the viability of cells, and is positively correlated with systolic blood pressure, diastolic blood pressure and urine protein	[157]
GHET1	placenta	downregulated	the E-cadherin / vimentin /fibronectin	GHET1 promotes PE by inhibiting cell proliferative, migratory, invasive capacities and EMT by stimulating E-cadherin and suppressing vimentin and fibronectin.	[158]
TDRG1	placenta	downregulated	miR-214-5p/Notch signaling pathway	TDRG1 inhibits cell viability, proliferation, migration, and invasion by stimulating the expression of miR-214-5p and regulating the Notch signaling pathway in trophoblast cells.	[159]
MALAT1	placenta	downregulated	pro-apoptotic proteins	Silencing of MALAT-1 in JEG-3 cells suppresses proliferation, migration and invasion, and induces cell cycle arrest at G0/G1 phase and enhancing apoptosis.	[160]
			N-cadherin/vimentin /E-cadherin/Hu-antigen R (HuR) /FOS	Down-regulated MALAT1 inhibits trophoblast invasion, migration, epithelial mesenchymal transition (EMT) and spiral artery remodeling by upregulating E-cadherin and downregulating FOS, N-cadherin, and vimentin.	[161]
			miR-206/IGF-1 axis	Down-regulated MALAT1 regulates miR-206/IGF-1 axis and thereby inhibits trophoblast cells migration and invasion through PI3K/Akt signal pathway.	[49]
TCL6	placenta	upregulated	PTEN/CDK2	Overexpression of lncRNA TCL6 is positively correlated with systolic blood pressure, diastolic blood pressure and urine protein, whereas negatively correlated with fetal birth weight of PE patients	[162]
uc003fir	placenta /preeclamptic placenta vessels/ vessel-free tissue	upregulated	CCL5/miR-155	Over-expression of lncRNA uc003fir increases proliferation, migration, and invasion of HTR-8/SVneo cells.	[163]
FOXD2-AS1	placenta	downregulated	miR-3127/MMP2/MMP9	FOXD2-AS1 silencing decreases the promotion effects on trophoblast cell induced by miR-3127 inhibition, partly mediated by influencing MMP2 and MMP9 levels.	[164]
KCNQ1OT1	placenta	downregulated	miR-146a-3p	KCNQ1OT1 could target the regulation of miR-146a-3p through CXCL12/CXCR4 pathway in the proliferation, invasion and migration of HTR8/SVneo cells.	[165]
SNHG5	placenta	downregulated	miR-26a-5p/N-cadherin	Knockdown of SNHG5 inhibits trophoblast (HTR-8/SVneo) cell proliferation, invasion, and migration, and promotes apoptosis and caused increase of cell population at the G 0 /G 1 phase and decrease of cell population at the S phase.	[166]
PVT1	placenta	downregulated	PI3K/AKT	PVT1 knockdown notably inhibits the proliferation, migration and invasiveness abilities of trophoblast cells, and significantly promotes the apoptosis through PI3K/AKT pathway.	[167]
			Ezh2/ANGPTL4	PVT1 knockdown notably inhibits cell proliferation and stimulates cell cycle accumulation and apoptosis by repressing ANGPTL4 transcription through binding with EZH2 in trophoblast cell.	[168]
WDR86-AS1	placenta	upregulated	miR-10b-3p / LITAF	WDR86-AS1 downregulates miR-10b-3p but promotes LITAF expression, which controls the inflammatory responses and migration and proliferation of HTR-8/SVneo cells under hypoxia.	[169]
ATB	placenta	downregulated	/	Down-regulated lncRNA-ATB decreased migration, proliferation, tube-formation of HTR-8/SVneo cells.	[170]
RPAIN	placenta	upregulated	C1q	The increased RPAIN levels may contribute to the development of preeclampsia through regulating trophoblast proliferation, invasion and apoptosis via C1q.	[171]
NR_002794	placenta	upregulated	/	NR_002794 has suppressive effects on proliferation and migration, and results in an increased rate of apoptosis.	[172]
MVIH	placenta	downregulated	Jun-B	The silencing of MVIH expression inhibits cell growth, migration, invasion, and angiogenesis in various trophoblast cell lines by modulating Jun-B protein expression.	[173]
GASAL1	placenta	downregulated	SRSF1/mTOR/EBP1/Bcl-2/caspase-3	lncRNA GASAL1 interacts with SRSF1 to regulate the proliferative, invasive, and apoptotic abilities of trophoblast cells via the mTOR signaling pathway.	[174]
MIR193BHG	placenta	upregulated	/	/	[175]
PROX1-AS1	placenta	upregulated	/	/	
GATA3-AS1	placenta	upregulated	/	/	
00511	placenta	downregulated	miR-31-5p / HOXA7	Down-regulated lnc00511 suppresses proliferation, invasion and autophagy, and enhances apoptosis in trophoblast cells to mediate pre-eclampsia progression through modulating the miR-31-5p/homeobox protein A7 axis.	[176]
	placenta	downregulated	miR-29b-3p/ Cyr61	AP2γ mediates downregulation of lncRNA LINC00511 as a ceRNA suppresses trophoblast invasion, proliferation and migration by regulating miR-29b-3p/Cyr61 axis.	[177]
AK002210	serum	downregulated	/	AK002210 knockdown suppresses trophoblast invasion, proliferation and migration	[178]
ZBTB39	placenta	upregulated	miR-210/THSD7A	Upregulated LncZBTB39 suppresses trophoblast invasion and migration via antagonizing the inhibition of miR-210 on THSD7A expression.	[179]
GAS5	placenta	upregulated	miR-21/MMP-9/TP53	GAS5 suppresses trophoblast invasion, proliferation and migration through the regulation of miR-21 and the activation of PI3K/AKT signaling pathway and its downstream proteins covering MMP-9 and TP53.	[180]
VIM-AS1	placenta	downregulated	E-cadherin/Snail/Vimentin	Down-regulated VIM-AS1 suppresses epithelial-to-mesenchymal transition (EMT) by upregulating E-cadherin and downregulating Snail and Vimentin.	[181]
FAM99A	placenta	downregulated	Wnt/β-catenin	Down-regulated FAM99A suppresses the invasive and migratory abilities of HTR-8/SVneo, and increases the apoptotic rate.	[182]
HIF1A-AS2	placenta	downregulated	LSD1/PHLDA1	HIF1A-AS2 suppresses trophoblast cell invasion and proliferation through upregulating PHLDA1 expression.	[183]
00261	placenta	upregulated	miR-558/TIMP4	Lnc00261 exerts the suppressive effects on the trophoblast invasion and migration via targeting miR-558/TIMP4 axis, which may involve in the pathogenesis of PE.	[184]
Uc.294	placenta	upregulated	/	Uc.294 inhibits proliferation, invasion and promotes apoptosis of trophoblast cells HTR-8/SVneo.	[185]
PSG10P	placenta	upregulated	miR-19a-3p/IL1RAP	IL1RAP promotes the expression of caspase-3 but inhibits MMP9 to suppresses proliferation, migration, and invasion of trophoblast cells.	[186]
00473	placenta	downregulated	LSD1/TFPI2	lnc00473 inhibits the expression of tissue factor pathway inhibitor 2 (TFPI2) through binding to lysine-specific demethylase 1 (LSD1) to inhibits cell proliferation and promotes apoptosis.	[187]
Uc.187	placenta	upregulated	PCNA and Ki67/MMP-2/−9 / TIMP-1/ Bcl-2	Uc.187 suppresses cell proliferation and invasion and promotes the cellular apoptotic response	[188]

Although there is no report that the methylation level of lncRNA itself is related to PE, abnormally expressed lncRNAs can regulate the proliferation, invasion, and apoptosis of trophoblasts by regulating the methylation of downstream molecules. Zhao et al. found that when lncRNA HOTAIR is expressed at high levels, it targets miR-106 by binding to EZH2 [139], which in turn inhibits the transcription of the target gene by inducing H3K27 methylation in the promoter region, ultimately suppressing the proliferation, migration, and invasion of trophoblasts [190]. Xu et al. further confirmed that EZH2 can directly interact with the promoter region of RND3 by methylating H3K27, the 27th amino acid of histone H3, thereby reducing the expression of RND3 in PE. However, the downregulation of lncRNA TUG1 in placental tissues inhibits the proliferation, invasion, and migration of trophoblasts and promotes their apoptosis and it also obstructs spiral artery remodeling by reducing the transcriptional regulation of RND3, which is mediated by recruited EZH2 proteins [143]. Li et al. reported, for the first time, that TUG1 acts as a molecular sponge for miR-29b, thereby regulating the expression of MCL1, VEGFA, and MMP2; it is therefore involved in the development of PE [14]. Many studies have shown that H19 mutations are closely related to PE. Moreover, lncRNA H19 is upregulated in PE. This activates the PI3K/AKT/mTOR pathway, which reduces trophoblast activity and increases invasion and autophagy [153]. Zuckerwise et al. also proposed that the downregulation of H19 inhibits TGF-β signaling transduction by reducing the Par6/Smurf1/RhoA pathway activated by TβR3 [152], Gao et al. found that this impairs the migration and invasion of extravillous trophoblasts in vitro by upregulating the expression of miR-let-7 and downregulating the expression of miRNA-675 [150]. TβR3 is considered to be a downstream mediator of H19–let-7 interaction. Zhou et al. demonstrated that H19 alters genome-wide methylation levels by regulating the activity of *S*-adenosyl-l-homocysteine hydrolase, and H19 knockout in the intron region causes the undermethylation of TβR3 [191]. Dey et al. showed that hypermethylation of the H19 promoter is associated with reduced H19 expression [192]. Furthermore, the absence of H19 imprinting in PE placental tissues reduces the invasive ability of trophoblasts, and may be associated with severe hypertension, which exacerbates PE [193].

### Diagnostic value of LncRNAs in PE

Table 5 reveals that lncRNAs may serve as potential diagnostic biomarkers for PE through ROC curve analysis. In placenta, Wu et al. have found that ROC of lnc TCL6 could reach to 0.8625 [138]. The ROC values of lnc BC030099 in the whole blood cells were 0.713 [104] and that of serum lnc AF085938, and that of lnc G36948 and lnc AK002210 were 0.7673, 0.7956 and 0.7575 respectively [178].

**Table 5:** Diagnostic value of lncRNAs in PE

LncRNA	Sample type	Area under curve	Sensitivity	Specificity	Ref.
BC030099	the wholel blood	0.713	/	/	[104]
NR_026824.1	the wholel blood	0.594	/	/	[104]
AK055151.1	the wholel blood	0.512	/	/	[104]
NR_027457	the wholel blood	0.532	/	/	[104]
NR_024178	the wholel blood	0.542	/	/	[104]
TCL6	placenta	0.8625	/	/	[138]
MIR193BHG	placenta	0.819	/	/	[159]
GATA3-AS1	placenta	0.640	/	/	[159]
PROX1-AS1	placenta	0.690	/	/	[159]
NR_027457	serum	0.5633	/	/	[178]
AF085938	serum	0.7673	/	/	[178]
G36948	serum	0.7956	/	/	[178]
AK002210	serum	0.7575	/	/	[178]

### Association between lncRNA variants and the risk of PE

Few studies have been conducted on the relationship between lncRNA gene polymorphisms and PE susceptibility. There is a significant correlation between the HOTAIR rs4759314AG genotype and higher PE risk, and the HOTAIR rs10783618 polymorphism is associated with increased PE risk in recessive and allele models. However, HOTAIR gene polymorphisms rs12826786, rs920778, and rs1899663 are not associated with PE susceptibility. The CTGAC haplotype is associated with decreased risk of PE, whereas the CTGAT haplotype is associated with increased risk [194].

## CircRNAs and preeclampsia

CircRNAs are covalently closed circular ncRNA molecules. They are resistant to degradation by nucleic acid exonucleases because they lack a 5′ terminal with a cap and a 3′ terminal with a poly(A) tail. These characteristics enable circRNAs to fulfill many biological functions, such as acting as molecular sponges for miRNA, regulating gene transcription and translation, and binding to RNA-binding proteins [195].

### Expression pattern of circRNAs in patients with PE

In recent decades, researchers have confirmed that circRNAs are involved in a variety of diseases [196]. However, there have been few studies on the role of circRNAs in the pathogenesis of PE. Ou et al. discovered 49 differentially expressed circRNAs in the placental tissues of patients with severe PE, using RNA sequencing, and further verified the upregulated expression of hsa_circ_0001438, hsa_circ_0001326, and hsa_circ_32340 by quantitative PCR analysis. To determine the interaction between circRNAs and miRNAs, they conducted an analysis of the Kyoto Encyclopedia of Genes and Genomics database. They found that the MAPK signaling pathway was the most enriched pathway in terms of circRNAs, and that the circRNA–miRNA–mRNA interaction network generated by hsa_circ_0001438, hsa_circ_0001326, and hsa_circ_32340 might be involved in the pathogenesis of PE. They also found that miR-145-5p was closely associated with circRNAs and mRNAs [197]. Table 6 generalizes the aberrant expression of circRNA.

**Table 6:** Dysregulation of circRNAs in PE

CircRNA	Sample type	Status	Function	Ref.
hsa_circ_0001438	placenta	upregulated	hsa_circ_0001438, hsa_circ_0001326, and hsa_circ_32340 were upregulated in the sPE patients and the circRNA-miRNA-mRNA interaction network generated with these three circRNAs revealed a broad regulatory network that might be involved in the pathogenesis of sPE	[197]
hsa_circ_0001326	placenta	upregulated		
hsa_circ_32340	placenta	upregulated		
hsa_circ_101,222	placenta	upregulated	Plasma protein endoglin in combination with circ-101,222 strengthened the predictive power for pre-eclampsia	[198]
hsa_circ_SFXN1	placenta	upregulated	CircSFXN1 overexpression significantly inhibited the invasion of TEV-1 trophoblasts and blocked the angiogenesis of human umbilical vein endothelial cells	[199]
hsa_circ_0011460	placenta	upregulated	Circ-0011460 were involved in vasodilation, regulation of blood vessel size, protein transport and localization	[200]
hsa_circ_0088227	placenta/plasma	downregulated	Knockdown of circPAPPA led to decreased proliferation and invasion in HTR8-S/Vneo trophoblast cells	[13]
hsa_circ_TNRC18	placenta	upregulated	Circ-TNRC18 enhanced trophoblast cell migration and epithelial-mesenchymal transition	[15]
hsa_circ_0014736	placenta	upregulated	the three altered circ-RNAs had a relationship with transcription regulation, proliferation, protein binding, and response to hypoxia	[201]
hsa_circ_0015382	placenta	upregulated		
hsa_circ_0007121	placenta	downregulated		
hsa_circ_100782	placenta	upregulated	/	[202]
hsa_circ_102682	placenta	upregulated	/	
hsa_circ_104820	placenta	upregulated	/	
hsa_circ_0001855	placenta	upregulated	Circ-0004904 and circ-0001855 combined with PAPP-A might be promising biomarkers for the detection of PE	[203]
hsa_circ_0004904	placenta	upregulated		
hsa_circ_3286	placenta	downregulated	Circ-3286 significantly promoted HTR8/Svneo cell invasion.	[204]
hsa_circ_593	placenta	downregulated	/	
hsa_circ_3800	placenta	downregulated	/	
hsa_circ_3286	plasma	downregulated	/	
hsa_circ_0036877	plasma	upregulated	Circ-0036877 significantly increased apoptosis of syncytial trophoblasts in the PE placenta	[205]
	placenta	downregulated		
hsa_circ_0036878	placenta	downregulated	/	
hsa_circ_0055724	placenta	downregulated	/	
hsa_circ_0049730	placenta	downregulated	/	
hsa_circ_0036474	placenta	upregulated	/	

### Diagnostic value of circRNAs in PE

In recent years, numerous studies have confirmed the value of plasma circRNAs as potential early biomarkers of PE. Using quantitative reverse transcription PCR, Hu et al. demonstrated that the levels of circ-0036877 in blood samples taken from patients with PE were significantly higher than those in the control group. Furthermore, ROC curve analysis suggested that plasma circ-0036877 is a potential early biomarker of PE: the area under the curve value was 0.846, the sensitivity was 85.3%, and the specificity was 72.7% [205]. Zhang et al. first published reports on the analysis of circRNA expression in blood cells. Analysis of red blood cell samples taken from 32 patients with PE and 32 healthy pregnant women revealed significantly higher circ-101,222 levels in patients with PE than in the healthy women: the area under the ROC curve, the sensitivity, and the specificity were 0.706, 65.61, and 68.54%, respectively [198]. To further verify the value of protein-bound circRNAs in the early diagnosis of PE, Bai et al. combined the plasma protein ENG with circRNAs. They found that the resulting area under the curve value increased to 0.876 (95% confidence interval (CI): 0.816–0.922), sensitivity increased to 70.73%, and specificity increased to 80.49%, compared to the corresponding values for the unbound circRNAs [201]. Table 7 summarizes the reported results.

**Table 7:** Diagnostic values of circRNAs in PE

CircRNA	Sample type	Area under curve	Sensitivity	Specificity	Ref.
hsa_circ_101,222	placenta	0.706	65.61%	68.54%	[198]
hsa_circ_0007121	placenta	0.72	77%	70%	[201]
hsa_circ_100782	placenta	0.653	/	/	[202]
hsa_circ_102682	placenta	0.774	/	/	
hsa_circ_104820	placenta	0.995	/	/	
hsa_circ_0001855	placenta	0.621	53.33%	70.00%	[203]
hsa_circ_0004904	placenta	0.611	/	/	
hsa_circ_3286	placenta	0.764	80%	68.60%	[201]
hsa_circ_0036877	plasma	0.846	85.30%	72.70%	[205]

## Relationship between lncRNAs and miRNAs

**Fig. 1:**
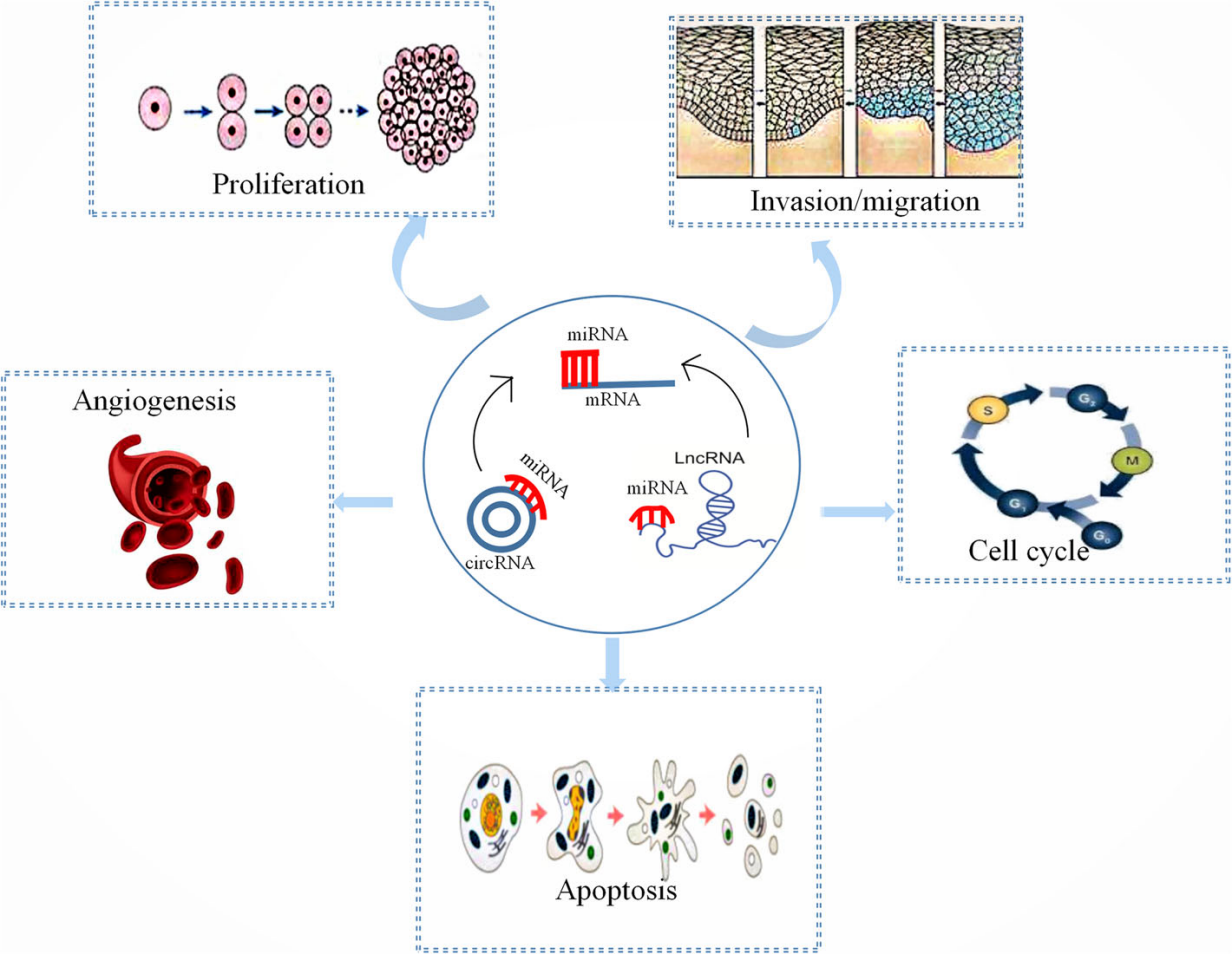
lncRNAs and circRNAs can act as a competing endogenous RNA(ceRNA), binding with miRNAs and regulating the effects of miRNAs on target genes, to influence trophoblast cell proliferation, invasion, migration and apoptosis and might play a role in angiogenesis and cell population at the G 0 /G 1 phase

As discussed above, both lncRNAs and miRNAs have regulatory effects on the pathogenesis of PE. Moreover, they are interrelated and interactive. lncRNAs can act as competing endogenous RNAs to influence the bioavailability of miRNAs [9]. Gao et al. first reported that H19 promoted the expression of miR-let-7 and downregulated miRNA-675, which resulted in the migration and invasion of extravillous trophoblasts in vitro [150]. Zuckerwise et al. also showed that the downregulation of H19 suppressed the Par6/Smurf1/RhoA pathway activated by TβR3 to reduce TGF-β signaling. TβR3 is considered a downstream mediator of the interaction between H19 and let-7 [152]. There have been numerous investigations into the role of the lncRNA–miRNA–mRNA axis in the pathogenesis of PE. For example, Tan et al. reported that DLX6-AS1 lncRNA may contribute to PE by suppressing the proliferation, migration, and invasion of trophoblasts via the miR-376c–GADD45A axis [155]. Li et al. were the first to report that lncRNA TUG1 causes the development of PE by acting as a molecular sponge for miRNA-29b, thereby regulating the expression of MCL1, VEGFA, and MMP2 [14]. Yu et al. discovered that lncRNA TUG1 can also act as a molecular sponge for miR-204-5p, and downregulated lncRNA TUG1 suppresses trophoblast migration and invasion, partly by sponging miR-204-5p [144]. Figure 1 depicts the molecular mechanism by which ncRNA affects the pathogenesis of PE.

## Relationship between circRNAs and miRNAs

**Fig. 2:**
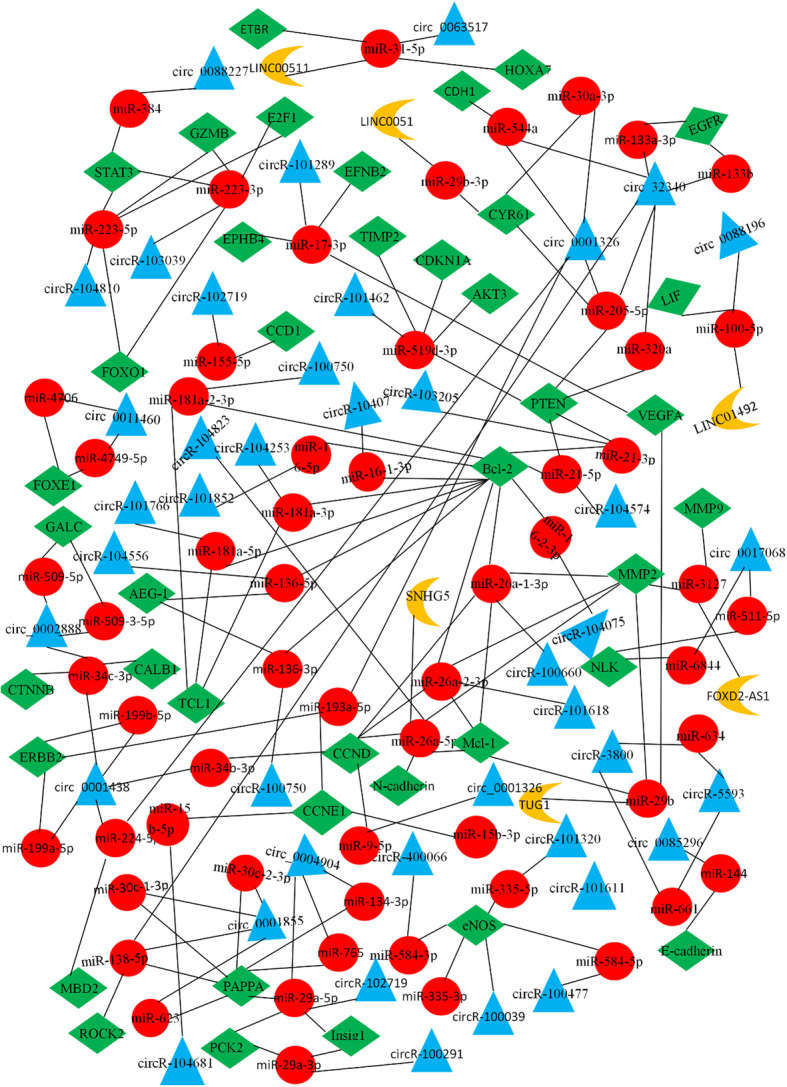
Mechanisms of interaction of ncRNA in PE. Green: protein coding, Yellow: lncRNA, Red: miRNA, Blue: CircRNA

CircRNAs that contain miRNA response elements can serve as competing endogenous RNAs by binding with miRNAs. They act as miRNA sponges in cells, thereby regulating the effects of miRNAs on target genes and altering their expression levels [9]. In 2013, Hansen et al. found that ciRS-7, which is a circRNA sponge for miR-7 derived from the *CDR1* gene, can bind and adsorb miR-7, thereby reducing its activity and indirectly upregulating the expression of miR-7-related target genes [206]. circRNAs have a stronger potential to adsorb miRNAs in the body than linear mRNAs or lncRNAs, because they are more stable. A few researchers have reported the occurrence of PE involving the interaction between circRNAs and miRNAs. circ-PAPPA, which is downregulated in both the placentas and plasma of patients with PE, can directly target miR-384 and act as a sponge for it. Finally, miR-384 overexpression inhibits the proliferation and invasion of trophoblasts by targeting STAT3 [13]. Shen et al. reported that upregulated circ-TNRC18 in the placental tissues of patients with PE also combined with miR-762 to target GRHL2 protein to regulate trophoblast epithelial–mesenchymal transition and invasion [15]. Although the role of circRNAs in the pathogenesis of PE is not fully understood, there is new evidence that they act as molecular sponges for miRNAs. Figure 2 illustrates the relationships between the various ncRNAs.

## Discussion

The heterogeneity and complexity of PE make its diagnosis, prediction, and treatment difficult. As it is currently not possible to detect the molecular signature of the main affected organ, i.e., the placenta, until the termination of pregnancy, it is difficult to monitor the progression of PE in a timely manner. Therefore, markers that circulate in the peripheral blood have great potential for noninvasive monitoring. It is currently possible to detect numerous biochemical markers in the serum, such as placental growth factor, soluble FMS-like tyrosine kinase receptor 1, placental protein 13, and placental protein A; however, their sensitivity and specificity are low [207]. Molecular biomarkers could provide a more reliable platform for the screening and diagnosis of PE than biochemical markers. In particular, the ncRNAs in the maternal peripheral blood are expected to be useful noninvasive biomarkers.

The differential expression of ncRNAs has been investigated to confirm their involvement in the pathogenesis of PE. Following numerous studies on the abnormal expression of ncRNAs in placental tissues, studies have been carried out to investigate ncRNAs in the peripheral blood of patients with PE. For example, Li et al. separated exosomes from maternal plasma by continuous density gradient hypercentrifugation, and found that seven miRNAs were differentially expressed in the exosomes from women with PE and those from a control group; however, the source of these exosomes was not determined [208]. It has subsequently been reported that exosomes derived from human umbilical cord mesenchymal stem cells that overexpress miR-139-5P, can promote trophoblast migration, invasion, and proliferation, and prevent apoptosis [209]. Studies on placenta-specific miRNA clusters in plasma samples revealed that the overexpression of miR-517-5p, miR-518b, and miR-520 h was associated with the late development of PE, and the screening of plasma miR-517-5p in early pregnancy also identified a proportion of women with subsequent PE [124]. Furthermore, Sun et al. performed univariate and multivariate analyses on the upregulation of lncRNA BC030099 in the whole blood of patients with PE, and determined that lncRNA BC030099 was an independent predictor of PE [137].

LncRNAs can regulate miRNA activity, and post-transcriptional regulation will affect the expression and function of their target mRNAs. LncRNAs have been shown to have miRNA binding sites --miRNA responsible elements, and they may potentially sponge the miRNAs, Thus, miRNA-mediated post-transcriptional regulation of target mRNAs was impaired. Dong et al. have demonstrated that LINC00511 regulates the proliferation, apoptosis, invasion and autophagy of trophoblast cells to mediate PE progression through modulating the miR-31-5p/homeobox protein A7 axis through dual luciferase reporter gene analysis [176]. When circRNAs interacted with miRNAs, they formed miRNA molecular sponges that further inhibited the transcript and lead to gene silencing. Due to the complementarity between bases, miRNAs bound to target mRNAs and performed transcriptional silencing to regulate gene expression. However, Li et al. confirmed circ_0063517 acts as ceRNA,targeting the miR-31-5p-ETBR axis to regulate angiogenesis of vascular endothelial cells in PE by dual luciferase reporting system and RNA immunoprecipitation (RIP) analysis [210]. Because lnc00511 and circ0063517 played an important role in the occurrence and development of PE through the bridge relationship of miR-31-5p, we therefore darw the conclusion that circRNA was associated with lncRNA through miRNA. In addition to that, lnc00511 functioned as a molecular spong for miR-29b-3p,antagonizing its ability to repress Cyr61 protein translation, and meanwhile overexpression of lnc00511 promoted trophoblast cell proliferation, migration and invasion [177].It is through this network that miRNA, lncRNA and circRNA are inseparable and jointly promote the occurrence and development of PE.

Although numerous studies have confirmed the differential expression of ncRNAs in placental tissues, and their pathogenic mechanism in PE, studies on ncRNAs in the peripheral blood, especially circRNAs and lncRNAs, remain scarce. More research is required to elucidate the key role of ncRNAs in PE, because they are potential stable biomarkers for the diagnosis of the disorder.

## Conclusions

The present review summarizes the expression patterns of ncRNAs, i.e., microRNAs (miRNAs), long noncoding RNAs (lncRNAs), and circular RNAs (circRNAs), and the mechanisms by which they affect PE. We examine the clinical significance of ncRNAs as biomarkers for the diagnosis of PE, and discuss the contributions made to PE by genetic polymorphisms and epigenetic ncRNA regulation. We believe that our study makes a significant contribution to the literature because it highlights the clinical value of ncRNAs as noninvasive biomarkers of PE.

## Data Availability

Datasets are available through the corresponding author upon reasonable request.

## Abbreviations

CircRNA: Circular RNA
LncRNA: Long noncoding RNA
MSCs: Mesenchymal stem cells
MiRNA: MicroRNA
NcRNA: Noncoding RNA
PiRNA: Piwi-interacting RNA
PE: Preeclampsia
ROC: Receiver operating characteristic
SNP: Single nucleotide polymorphism
SiRNA: Small interfering RNA
SnRNA: Small nuclear RNA
